# Characteristics of the Outdoor Environment Affording Physical Activity, Motor Competence, and Social Interactions in Children Aged 3–7 Years: A Systematic Review

**DOI:** 10.3390/children11121491

**Published:** 2024-12-06

**Authors:** Nicola Taylor, Andy Pringle, Clare M. P. Roscoe

**Affiliations:** Clinical Exercise Rehabilitation Research Centre, School of Sport and Exercise Science, University of Derby, Derby DE22 1GB, UK; a.pringle@derby.ac.uk (A.P.); c.roscoe@derby.ac.uk (C.M.P.R.)

**Keywords:** physical activity, play, outdoors, intervention, children, early childhood education, motor competence

## Abstract

Early childhood education (ECE) settings play a crucial role in promoting physical and social development among children aged 3–7 years. This systematic review sought to examine the associations between characteristics of ECE outdoor environments, social interactions, physical activity, and motor competence. The secondary aim examines previously applied methods to capture children’s behaviour in the context of their social and physical environment. Methods: This review used the PRISMA framework and study quality was assessed using the mixed-methods appraisal tool (MMAT). Keyword searches were conducted in seven databases. Studies were eligible if children were aged 3–7 years in ECE; physical activity, social interactions and/or motor competence were measured; location and/or social context were measured. Results were synthesised using an effect direct plot, a table of associations, and narrative synthesis. Results: Twenty-three studies from eight countries met the inclusion criteria. Intervention and controlled cross-sectional studies (*n* = 9) favoured high-quality outdoor environments rich in affordances, portable play equipment, and natural features to increase children’s physical activity, social interactions, and cooperative play. Cross-sectional and descriptive studies (*n* = 14) positively associated open grassy space, portable and fixed equipment, wheeled toys, and paths with physical activity (*p* < 0.05). Based on limited evidence, playground size and active games in small groups were associated with greater MC. Conclusions: The findings highlight the benefit of creating diverse affordance rich outdoor environments in early childhood settings to promote physical and social development. Limitations include variability in study designs and protocols for conducting systematic observations, thus emphasising the need for standardised approaches to future research.

## 1. Introduction

Physical activity (PA) and opportunities for regular outdoor play are essential in early childhood, promoting healthy development of cognitive, physical, emotional, social, and motor skills, as well as overall well-being [1,2]. PA and motor skill development are particularly important during early childhood, building the foundations for lifelong health and active living [3]. Consequently, the World Health Organisation (WHO) recommends at least 180 min of physical activity per day for children aged 5 years and under and children aged over 5 years should aim to achieve 60 min of moderate-to-vigorous PA (MVPA) per day [4]. Attaining adequate levels of MVPA can promote a range of health benefits including the development of a child’s motor competence (MC), and being proficient in a range of motor skills is vital for children to develop sport-specific skills and promote physically active lives that span across the life-course [5]. Consequently, it is vital that early childhood experiences should be characterised by frequent opportunities to engage in PA and active play in suitable child-friendly environments to ensure the healthy development of young children.

Despite the importance of adequate PA and early proficiency of MC, it is concerning that many preschool children do not meet the PA guidelines, leading to increasingly sedentary lives [6,7]. Globally, it is estimated that 40% of preschool children do not adhere to the guidelines [4], while 88.9% of 2–4-year-old children were failing to meet the recommended 180 min/day in England [6]. The recent UK Active Lives survey reports that over 50% of over 5′s are inactive, reflecting the decline in PA in children over time [8]. Further compounding the issue, children’s MC proficiency is also concerningly low [9,10,11], as children are not achieving a full range of movement competencies by the recommended age of 7 years [11]. Reasons attributed to this current state are likely to be multifaceted, but a shift towards sedentary indoor activities [12], restricted outdoor play time [12,13], and shifts in educators’ and parents’ attitudes to health and safety concerns are likely contributing factors [12,14,15]. Considering the wide-reaching benefits of PA, further interventions are required to establish positive health behaviours in the early years.

Given that most 4–5-year-old children in the UK are in formal childcare or school-based education, early childhood education (ECE) settings are crucial environments for promoting and supporting movement behaviours [16]. For instance, it is widely acknowledged that children are more active when playing outdoors (43.7%) compared to indoors (20%) [17] during the ECE day, and time spent outdoors is positively associated with PA and negatively associated with sedentary behaviours [18]. Nonetheless, conflicting research has demonstrated that increasing outdoor time alone may not be an effective invention for achieving the PA guidelines [19,20], as other factors may be more pivotal in promoting PA behaviours outdoors [21].

Considering that increasing time in the outdoors may have limited impact on increasing children’s PA, researchers’ have examined other outdoor variables such as the physical characteristics of the environment [22,23,24]. For example, previous reviews [22,23] demonstrate that introducing portable play equipment significantly increases time spent in light and vigorous PA while reducing sedentary time. Terrón-Pérez et al. [22] found positive associations between open spaces and PA, while fixed play equipment was equivocal, and the presence of soft ground surface materials was negatively associated with PA [22]. Similarly, Martin et al. [24] confirmed that availability of resources and sufficient size of outdoor play areas enable children to be physically active, and Johnstone et al. [25] found that certain natural features such as grassy hills and logs in children’s play spaces are more effective at promoting MVPA and motor skills such as balancing. However, a limitation reported in previous systematic reviews has been the quality of included studies, with most being uncontrolled cross-sectional designs. Despite alluding to associations between PA intensities and durations with physical environments, further interpretations are limited without contextual information [25].

The benefits of active outdoor play for young children’s development arguably extend beyond PA outcomes, as the environment affords opportunities for gross motor skill development through exploratory play behaviours with peers and through social interactions [2,26,27,28]. Hence, when examining the behaviours of young children, it is relevant to consider both the social and environmental context [29]. As such, social factors such as group size [30], gender, adult presence, and forms of cooperative play, in parallel with features of the environment, have been examined. In an earlier review by Trost’s et al. [23] social factors such as changes in educators’ practice were seen to relate to children’s movement behaviours in the outdoor context. Conversely, Larrea et al. [27] found that social interactions may be moderated by the provision of affordances, such as loose parts play where children cooperate to build and manoeuvre objects. Given that outdoor free play is an important feature in early years provision, educators are encouraged to facilitate child-led exploration of the outdoor space through movement and play [31]. Fulfilling this role requires careful consideration of the risks pitted against the benefits for learning and development. Hence, the educator can be seen as a gatekeeper of movement behaviours, illustrated in studies where child-initiated activities were positively associated with MVPA [21], or where the PA levels of the educator were deemed to predict the children’s activity levels [21,32]. Given that the environment comprises of both physical and social attributes, it makes little sense to separate them from each other. To further our understanding of children’s movement behaviours in an outdoor setting, it is necessary to consider these multiple factors. While previous reviews have acknowledged several associations between the physical environment and PA, there is less understanding as to how social interactions influence PA behaviours in the outdoor context.

To better understand the functional qualities of the outdoor environment that promote movement behaviours, numerous studies have adopted Gibson’s theory of affordances [33,34]. Affordances are described as possibilities for action that a child perceives in their physical environment and may actualise according to their individual capabilities [34,35]. It is postulated that outdoor environments afford more diverse opportunities for action that enhance motor skills and PA [36]. For instance, prior studies based on affordance theory report higher levels of PA and more diverse movement behaviours when children are exposed to environments rich in natural elements or loose parts [37,38,39,40]. These elements offer extensive open-ended movement challenges individualised by the child and encourage creativity and risk-taking play [41]. According to Johnstone et al. [25], these risk affordances may be highly attractive to children and be attributed to why the outdoors promotes more active play in children. As affordances are relational and are a product of the interaction between the environment and the individual, it is important to consider how prior research has captured these revelations [39]. Despite previous systematic reviews acknowledging the need for this information, there remains a gap in the literature whereby the methods for examining the context of children’s PA behaviours are reviewed. Research is therefore needed to further our understanding of effective mechanisms in ECE outdoor settings.

Capturing detailed contextual information presents several methodological challenges, most notably the need to establish reliable and valid observational tools that are feasible to administer. Due to the time-intensive nature of using direct observation, researchers are often required to capture adequate information in a short timeframe that is representative of participants’ behaviour. Previous approaches have included the use of self-report [42,43], alternatively direct observation [43], and mapping tools have been deployed thus synchronising behaviours and context. More recent advances in technology are increasingly being applied to capture data with greater ease, replacing direct live observations with video analysis, and use of integrated geographic information systems (GIS), global positioning systems (GPS), and accelerometers to map behaviours with locations [44]. Despite these advances, there remains a high degree of variability in the observational tools applied to accurately measure the movement behaviours, social context, and environmental characteristics. To advance the research in this field, it is necessary to review previous methods and the use of systematic observation to establish future research recommendations and standardised approaches.

Therefore, the aims of this review were the following:

1. Examine the relationships between the ECE outdoor environment, social interactions, physical activity, and motor competence of children aged 3–7 years.
2. Identify common observational tools and methods of measuring PA behaviours in the context of the physical and social environment.

## 2. Materials and Methods

### 2.1. Protocol and Registration

This systematic review was conducted according to the Preferred Reporting Items for Systematic reviews and Meta-Analysis (PRISMA) and was registered with PROSPERO (registration number CRD42023488731) in December 2023. The review protocol can be found by searching this number on the PROSPERO website or using the address https://www.crd.york.ac.uk/prospero/display_record.php?RecordID=488731 (accessed on 11 November 2024). For this review’s purposes, both inactive and active unstructured play were considered to examine the relationship between the characteristics of the outdoor environment and physical and social outcomes.

### 2.2. Study Selection Criteria

To be eligible for this review all English language peer reviewed articles published between 2010 and 31 January 2024 were included, this allowed for a more focused review reflecting current methodological advancements in technologies as well as reflecting the release date of the WHO physical activity guidelines including children [45]. Database searches included observational studies, prospective cohort studies, intervention, and validity studies. Review articles, conceptual, and qualitative studies were excluded for analysis.

The literature included in this review met the following eligibility criteria: (a) participants between the ages of 3–7 years, (b) the intervention or comparator required the use of the ECE outdoor play space, (c) examined PA measured by either accelerometry or systematic observation, and/or motor skills, and/or social interactions, (d) measures must include either observations or tracking devices to capture the social or environment context, (e) the outcomes were measured during a typical day at childcare or preschool, (f) the observations were collected during recess or unstructured periods of free play, and (g) data were collected from typically developing and typical weight children without disability or developmental delay. Studies were excluded if the participants were outside of the 3–7 years age group. Children aged 3–7 years were chosen because this group would typically attend ECE, the 3–7 years range accounts for global differences in the ages of children attending ECE provision [25].

### 2.3. Search Strategy

The literature searches were conducted on the 31 January 2024, the following databases were searched for English articles: PubMed, Medline, EBSCO British Education Index, ProQuest, ScienceDirect, PsycINFO, and Scopus to ensure comprehensive coverage of the interdisciplinary literature on children’s PA and outdoor play. These databases were selected for their relevance to health, education, psychology, and environmental research, providing a diverse range of peer-reviewed publications. We identified three core concepts which formed the basis for the search algorithm, these were outdoor environment; preschoolers; and physical and social outcomes. For each concept, synonyms were listed and then converted into a search string for each database. Table 1 shows an example search string used for PubMed database.

**Figure 1:**
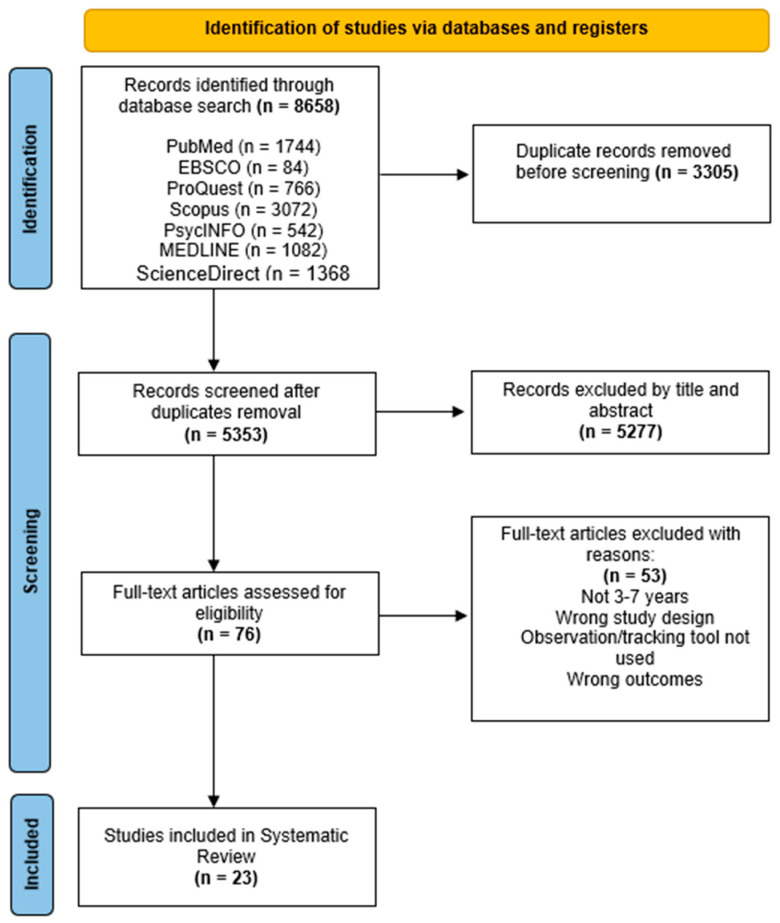
PRISMA flow diagram for systematic review.

The entire search was complete on 31 January 2024 and records were exported into Rayyan [rayyan.ai] a web-based software for screening selected data [46,47]. After detected duplicates were checked and removed, titles and abstracts were screened and articles not meeting the inclusion criteria were removed. Full-text articles were then reviewed for eligibility by the lead author, Figure 1 presents a visual representation of the search strategy. Ten percent of the final sample was randomly allocated and screened by a second researcher (CR), confirming eligibility of all articles.

### 2.4. Data Extraction and Synthesis

Data extraction was conducted by the lead author using Microsoft Excel to record the following information from each selected study: (a) first author and year; (b) country of origin; (c) sample size and sex; (d) participant age; (e) study objective; (f) research design; (g) environment variables and measurement; (h) outcomes measured and measurement tools (the table in Section 3.4). Findings for each study were then grouped and presented according to outcome and study design.

Outcomes were grouped into three categories: physical activity, motor competence, and social interactions. As a meta-analysis could not be performed due to studies being heterogeneous, a synthesis without meta-analysis based on effect direction was conducted for PA outcomes of intervention studies (*n* = 9); these outcomes were sedentary behaviour (SB), MVPA, and total physical activity (TPA). For each outcome, studies were grouped, and results represented by an effect direction plot providing a visual summary of overall effects of conceptually similar ECE outdoor interventions (the first table in Section 3.5). Associations between features of the outdoor environment and PA levels were grouped and represented in a simple vote count table (he second table in Section 3.5), associations were counted if statistical significance of at least < 0.05 was attained. Additionally, the summary of the table in Section 3.6 presents key findings from all studies that examined the ECE outdoor environment, PA, and social interactions, and the table in Section 3.7 provides a summary of study findings that reported a MC outcome. Narrative synthesis offers further insights into the intricacies of the effects and associations. Subgroups analyses allowed the effect of gender to be examined across all categories. Finally, this review also examined the procedures and reported reliability and validity properties of the observational tools used in studies to measure physical and social behaviours in the eligible studies (the table in Section 3.8).

### 2.5. Quality Assessment

The mixed-methods appraisal tool (MMAT) was used to assess the quality and risk of bias within the studies included in this review. The studies were screened using two initial questions before being appraised using five criteria according to their study design. The study designs in this review included quantitative randomised controlled trials, quantitative non-randomised and quantitative descriptive studies. To report the score for each study, we used a 5-star system whereby each criterion equated to a star, therefore a 5-star rating would indicate all criteria were met [48]. Of the 23 studies included in this review, 15 were of 5-star quality, 3 studies were 4-star quality, and the remaining 5 studies were between 1 and 3 stars (see Appendix A, Table A1 for full MMAT criteria and study scores).

## 3. Results

### 3.1. Search and Selection of Studies

The database search yielded 8658 articles. Duplicates were initially detected by Rayyan and then reviewed before a final decision was made to retain or remove each article, leaving 5353 articles [4,46]. After title and abstract screening, 76 articles were eligible for full-text screening, after which 23 articles remained for this review (see Figure 1). The most common reasons for exclusion were the wrong age of participants, absence of tracking device or systematic observation tool, and wrong study design or outcomes.

### 3.2. Origin and Participants

Table 2 presents the main characteristics of the selected articles. Of the 23 articles, the majority were conducted in United States (*n* = 10), Canada and Norway (*n* = 3), Australia and Portugal (*n* = 2); the remaining studies were conducted in Slovenia, the UK, and Japan (*n* = 1). The total number of participants was 1254 across the 20 studies that reported participant sample size. The remaining three studies reported the number of participating ECE centres along with the number of observations per centre. Sex differences were reported in 73% (17) studies (boys *n* = 574, girls *n* = 520). Sample sizes were small across studies; only two studies had a sample size of greater than 200, these were randomised control trials and cross-sectional studies. Socioeconomic status was only reported occasionally, and in most cases, this was moderate to high status.

### 3.3. Study Designs

Most study designs were uncontrolled cross-sectional (*n* = 11), fewer were controlled cross-sectional (*n* = 4), uncontrolled before and after (*n* = 5), and the remaining two studies were randomised control trials (*n* = 2) and descriptive (*n* = 1).

### 3.4. Study Quality Assessment

The quality of all included studies was assessed independently at study level by one reviewer (NT) using the MMAT. No studies were excluded due to the low quality, but issues were considered when interpreting the findings. According to the MMAT 65% (15 articles) of the studies met all criteria and were rated 5 stars, 13% were rated as 4 star (3 articles), and the remaining 5 articles were 1–3 stars. A full breakdown of the quality assessments and study scores can be found in Appendix A (Table A1).

### 3.5. ECE Outdoor Environment and Physical Activity

Physical activity was measured in 20 of the eligible studies. Actigraph accelerometers were used in 7 studies [50,51,52,62,67,69,76], 13 studies used a systematic observation tool [38,49,50,51,53,54,55,56,58,59,61,62,66], and 6 studies adopted both approaches [50,51,62,67,69,76]. Across the 13 studies that used systematic observation tools, 4 pre-validated tools were used, these were the Observational System for Recording Physical Activity in Children- Preschool (OSCRAC-P) [77], System for Observing Play and Leisure Activity in Youth (SOPLAY) [78], the System for Observing Children’s Activity and Relationships during Play (SOCARP) [79], and the Children’s Activity Rating Scale (CARS) [80]. Systematic observations were either conducted live through direct observation, video recorded for post analysis, or a combination of both methods (see the table in Section 3.8 for summary).

The eligibility criteria for this review required studies to measure the context and/or location of PA behaviours. Most studies achieved this by using systematic observation tools, alternative methods included combining an Actigraph wGT3X-BT accelerometer with a QStarz BTQ1300ST Global Positioning System [52]. Two studies used proximity sensing Radio Frequency Identification Devices to measure the social interactions of children [60,73]. All remaining studies used systematic observational tools, often in combination with behaviour mapping techniques to record physical and social behaviour in respect to their locations. Mapping software such as Geographic Information Systems (GIS) [54,66] and ArcMap 10.5.1 [52] were used to map predefined zones of the ECE outdoor play space and to analyse and visualise spatial data. Alternatively, maps were created manually using Adobe Illustrator [50], or free hand [56] or details were not reported.

In this review, intervention studies (*n* = 9) were all based on either renovating or reconfiguring the ECE play space to improve the environmental quality, either by introducing or increasing the availability of affordances; introducing natural elements; and/or increasing the availability of risk affordances to the play space [50,51,55,62,69,76]. Of these intervention studies, two measured SB [62,67], five included a measure of MVPA [38,50,51,62,76] and four measured total physical activity (TPA) [55,67,69,70]. In all three controlled cross-sectional studies, the natural environment was used as an exposure and the traditional ECE playground as comparator [38,67,70]. Table 3 presents the effect direction plot for SB, MVPA and TPA in eligible studies where these outcomes were reported in more than one study. Two studies demonstrated a significant decrease in time spent in SB postintervention. In one study, where the intervention was based on nature elements, a significant decrease in SB was observed at follow up in observation-based data (M_Diff_ = −25.6%, *p* < 0.001) [62], but this was not reflected in the accelerometer data for the same study. In the second study, Bundy et al. [51] observed an intervention effect following the introduction of recycled loose material to the playground (β = −2.1 min, 95% CI −3.77 to −0.51, *p* = 0.01) [51]. The intervention group were on average −2.1 min/ECE day less sedentary than children in the control group and without the recycled materials.

Time in MVPA was measured in five studies; two found significant increases where natural features and recycled affordances were used as intervention materials. Nicaise et al. [62] found children spent more time intervals in MVPA at postintervention (M_Diff_ = +14.9%, *p* < 0.001), as did Bundy et al. [51] who found a significant increase of 1.8 min/ECE day for the intervention group (95% CI 0.52 to 3.12, *p* = 0.006). Bjørgen’s [38] descriptive data demonstrated that children spent more time in moderate to high PA levels (>1 h/ECE day) when in a natural environment compared to the kindergarten play space. In contrast, Webster et al.’s [76] RCT study found no intervention effects on MVPA when painted stencils were introduced to the school playground. Furthermore, Brussoni et al. [50] demonstrated a significant decrease in MVPA (M_Diff_ = −1.32 min, SE = 0.37, < 0.001) when risky affordances were introduced to the outdoor space [50].

Four studies presented TPA, of these, three indicated an increase in TPA, but only two were statistically significant. These interventions were based on increasing the availability of affordances in the outdoor play space. Cosco et al.’s [55] renovation promoted movement by installing looped pathways and more vegetation, whereas Sumiya et al. [69] simply reconfigured the play space to increase the accessibility of playground affordances. Both studies demonstrated significant increases in device-measured TPA (*p* < 0.05, *p* < 0.001) at postintervention, with features such as tires, open areas, and tracks seen to increase PA levels compared to other features (*p* < 0.05). When studies compared children’s PA levels in traditional ECE playgrounds to natural environments, one study did not find any significant difference [67], and the remaining study observed an increase in distance (m) when children played in natural environments, although this was not statistically significant.

**Table 3:** Outdoor environment interventions on physical activity.

Study ID	Study Design	Sample Size	Intervention/Exposure Concept	MMAT	SB	MVPA	TPA
Cosco [55]	UBA	804	Renovation and natural affordances	4*			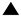
Brussoni [50]	UBA	45	Natural and risk affordances	5*		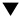	
Nicaise [62]	UBA	50/57	Reconfiguration and natural affordances	5*	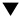	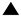	
Sumiya [69]	UBA	6	Reconfiguration and affordances	5*			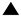
Webster [76]	RCT	51	Ground affordances	4*		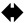	
Bundy [51]	Cluster RCT	221	Recycled material affordances	5*		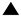	
Torkar [70]	CCS	25	Traditional vs. nature	3*			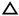
Storli [67]	CCS	16	Traditional vs. nature	3*	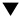		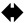
Bjørgen [38]	CCS	24	Traditional vs. nature	3*		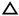	
	Summary Effect				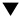	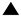	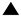

Abbreviations: UBA, uncontrolled before and after; RCT, randomised controlled trial; CCS, controlled cross sectional; SB, sedentary behaviour; MVPA, moderate to vigorous physical activity; TPA, total physical activity; MMAT, mixed-methods appraisal tool. Study level 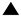 = significant mean increase from intervention/exposure; 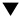 = significant mean decrease from intervention/exposure; 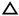 = non-significant increase from intervention/exposure; 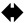 = no effect from intervention/exposure.

The remaining eight uncontrolled cross-sectional studies examined the associations between features of the ECE outdoor space and children’s PA behaviours. A summary of these associations (*p* < 0.05), together with those reported in the intervention studies are presented in Table 4.

Open spaces, grassy hills, and lower density areas were positively associated with PA [52,53,61,62,66] in all but two studies [63,66]. Portable equipment such as loose parts, balls, frisbees, tires, and wheeled toys were also positively associated with PA in seven studies [51,53,55,61,63,66,69]. However, some contradictory findings were elicited in Nicaise et al.’s [61] study, which examined two independent ECE playgrounds. For example, wheeled toys and fixed equipment were positively associated with PA in one school but no association was found in the second school, perhaps highlighting the significance of other contextual features in a setting, such as availability of equipment [52]. Furthermore, Clevenger et al. [52] demonstrated that fixed equipment could be a location of both high and low PA levels across the ECE day. Using spatiotemporal analysis, this novel approach reveals the effect of time of day on children’s preferences for activity and corresponding equipment. Another novel study examined the spatial layout of ECE playgrounds, Smith et al. [66] found that number of adjacencies and centrality of play settings were associated with PA levels. Finally, surface materials were found to be associated with PA, such as mulch and sand being negatively associated, along with hard surfaces and sidewalks being an attraction for social activity as reflected in Clevenger et al.’s [52] study.

### 3.6. ECE Outdoor Environment, Social Interactions, and Physical Activity

Of the 17 studies that reported social interactions, 9 were intervention studies or compared a traditional ECE playground to a natural environment to observe any changes in behaviour [38,50,51,55,56,60,67,70,76]. The remaining eight studies observed the associations between the features of the ECE outdoor space and social interactions, and/or social interactions when children were engaged in different PA behaviours [49,52,53,58,61,63,66,73]. Table 5 presents a descriptive summary of these findings.

Most studies (*n* = 14) measured social interactions via systematic observations, whereas a novel clustering technique combining GPS and accelerometers was used by Clevenger et al. [52], and Radio Frequency ID badges were used by Moreira et al. [60] and Veiga et al. [73] to measure social proximity. In addition to social interactions, one study measured social competence as an outcome measure [73]. In this review, cooperative play was also considered as an outcome indicating social interactions, and this was measured using bespoke systematic observation tools. Several techniques were employed to track children’s behaviour to location including systematic observations, behaviour mapping techniques, and use of GPS- and GIS-integrated systems.

The number of social interactions increased in studies where the natural environment was used as a comparator [38,56], and in intervention studies where affordances were introduced to the play space [50,51,60]. In contrast, Cosco et al.’s [55] study indicated that children’s activities post renovation were mostly alone; this behaviour likely reflects the nature of the renovation, being focused on looped pathways affording wheeled toy-based activities. Conversely, in studies where the environments were enriched with affordances and natural features, more diverse play, cooperative play, and peer activities were observed [38,50,51,56,60,70,76]. In two of the intervention studies, less teacher presence had a positive effect on the PA levels of children, and Cosco et al. [55] observed the negative effects of teacher custodial interactions on children’s PA levels. Interestingly, Brussoni et al. [50] noted postintervention, a reduction in teacher interactions due to the introduction of nature and risk affordances, and more diverse play exhibited by the children. Along with more peer play, two studies also observed more mixed gender play when in the nature-based or higher-quality environment [38,60], although there were no differences found in Brussoni et al.’s [50] study. Finally, in studies where gender differences were reported (*n* = 8), boys were more physically active than girls in all cases [53,55,60,61,63,66,70]. The exception to this was Veiga et al.’s [73] study, where more exercise play exhibited by girls was observed; however, boys demonstrated more time in rough and tumble play. It is therefore likely that boys were at least as active as girls in this study, but through different types of active play.

Cross-sectional studies (*n* = 8) reveal several similar and contrasting findings, for instance, both Smith et al. [66] and Connelly et al. [53] found that teacher presence was positively associated with MVPA, explained by individual teacher differences, and use of positive prompts. Across studies, PA was more likely when children were in small peer groups or alone, higher teacher to child ratios were also associated with higher PA intensities [49]. Veiga et al.’s [73] study found that the mean duration of social interactions was positively associated with social competence [73], and social competence was positively associated with exercise play. Similarly, Foweather et al.’s [58] findings suggest that children involved in active games in small groups had better motor competence compared to children less involved in these games. Despite children who were alone displaying more locomotor behaviours during recess, they exhibited lower motor competence overall, and specifically in locomotor skills.

### 3.7. ECE Outdoor Environments and Motor Competence

Motor competence was measured in three of the studies, one being an intervention study [76], and the remaining two were both uncontrolled cross-sectionals [58,72]. Movement competency was measured using the Children’s Health and Activity Motor Program Skills test (CHAMPS) in two studies [58,72] and the Test of Gross Motor Development (TGMD-3) in one study [76]. Table 6 presents a summary of these findings. One intervention study found no significant effects of painted ground stencils on total TGMD-3 scores of preschool children. Although there were significant mean changes in locomotor skills, ball skills and total TGMD-3 score in the intervention group, these were not statistically significant when compared to the control group. The remaining two studies were uncontrolled cross-sectionals and reported positive associations between the size of playground and motor competence scores (F(1, 159) = 4.30, *p* < 0.05, d = 0.33) [72], and time spent in active games without equipment was positively associated with total and locomotor FMS scores (Total FMS: β = 2.03, 95% CI 0.46–3.60, *p* < 0.05, locomotor skills: β = 1.08, 95% CI 0.23–1.93, *p* < 0.013) [58], and interestingly, children that spent more time exhibiting locomotor behaviours at recess were negatively associated with locomotor skills (β = −2.96, 95% CI −5.02–−0.89, *p* < 0.05) [58].

### 3.8. Systematic Observation Tools and Procedures

Across the 23 studies, 4 validated observation tools were used to measure children’s PA levels and, in most cases, contextual information regarding location, social interactions, and activity types (Table 7). Seven studies used OSRAC-P [38,53,59,61,62,63,72], three studies used CARS [54,55,66], one used SOPLAY [76], one study used the SOCARP tool [58], the remaining study used a combination of OSCRAC-P and SOPLAY [49]. Seven studies devised tools to capture play and social interactions of children’s behaviour [50,51,56,63,67,69,70,73]. One study inductively coded video footage to establish codes [69,75], whilst another coded play events by frequency and duration of engagement [75]. The remaining study used GPS and accelerometer data to locate clusters of high and low PA levels [52].

Studies that adopted pre-validated observation tools reported corresponding validation studies detailing psychometric properties. However, OSRAC-P is the only tool to have been validated specifically with preschool children in mind. SOPLAY, CARS and SOCARP were developed for older children and adolescent populations. Further, OSRAC-P is the most comprehensive tool that combines details on PA intensity, activity types, environmental context and social interactions. For establishing categories of play and social behaviours, five studies referred to the previous literature to inform categories for coding [50,56,60,63,70], but only one study reported prior piloting and reliability testing on the reported tool before application [56]. Although the previous literature was consulted, none of these tools had been through a formal validation process prior to use.

Reliability between raters was reported in 72% (*n* = 16) of the studies that used systematic observation, and values were all deemed acceptable [69]. Six of these reported Cohen’s Kappa coefficients and these ranged from 0.65 to 0.83. One article presented intraclass correlation coefficient > 0.75 and inter-rater agreement was reported in nine of the articles ranging from 80 to 99% agreement. The percentage of observations used for inter-rater reliability was provided in a smaller number of studies (*n* = 7, 31%), and even less (*n* =3, 13%) provided values for each category. Most studies (*n* =17, 77%) had more than 1 observer to code observations, and 12 studies (54%) reported observer training or procedures to maintain level of agreement between raters. Test–re-test reliability was only reported in two studies in this review. Of the 22 studies that used some form of observation, 9 (41%) conducted live observations, 10 (45%) used video, 2 (9%) used both live and video observations, and it was unclear how analysis was conducted in 1 study (5%).

Most studies (*n* =20, 90%) reported the items, categories, and any modifications made to the observational tool. In these instances, modifications used a combination of established tools, or by adopting categories reported elsewhere in the literature. One study established codes inductively via observing video footage and coding actions as they occurred, a second coder was deployed for consistency using the established codes and descriptors [69].

Across all studies, there was a high degree of variability regarding observation recording and sampling methods. As such, this review identified the following procedures across studies: (a) momentary time sampling; (b) partial interval recording; (c) one-zero-time sampling; (d) continuous recording of events and/or durations in an observational period. Observation intervals ranged from 5 s observation periods to 15 s in a 2 min period; other procedures coded events and durations continuously over periods ranging from 2 to 97 min. This review identified that observation procedures targeted either the individual child, group, or target zone, meaning that analyses were either conducted at the child level or in relation to predefined areas of the play space. Total number and duration of observations ranged from 50 to 11,825 and 87 to 665 min, respectively, illustrating high variability in observation samples for conducting systematic observation. Finally, observations were conducted at different times of the ECE day across studies, such as morning, lunch, and afternoon recess, and free play periods.

Of the articles reviewed, ActiGraph accelerometers were used in seven studies to objectively measure PA combined with observation tools for contextual information. Consistent with previous reviews, cut points and epochs were variable across studies, as was wear time, number of days of measurement, and what constituted as an acceptable day of wear. Issues concerning the high variability of these factors have been covered elsewhere in the literature [4,81,82], and as such they are outside the scope of the current review.

**Table 7:** Procedures and reporting of validity and reliability properties of observational tools across studies.

Observation Tool	Studies Used	Dimensions	Pilot Testing or Adaptations	Reliability Reporting	Validity Reporting	Video/Live Direct Observation	Observation Procedures	Observers (*n*)
CARS	Cosco [55]	PA intensity 1–5 contextual information. Other items: location, gender, social, teacher interactions.	Not reported	Cohen’s κ pre 0.719, post 0.832	yes	Direct live	6596 obs. Obs. zones	2
CARS	Cosco [54]	PA intensity 1–5. Other items: Gender, behaviour setting type and physical attributes.	Not reported	Not reported	yes	Direct live	101 min obs. Obs. zones	2
CARS	Smith [66]	PA intensity 1–5, duration and frequency, contextual information.	Trained observers in sub-analysis.	Interrater reliability Kappa coefficients 0.85, 0.71, 0.87, 0.70	yes	Direct live	6125 obs. Obs. zones. 7 min intervals 4 children per obs.	>1
SOPLAY	Webster [76]	Activity levels 1–3, gender, environmental factors, time of day, supervision, accessibility, organisation, equipment.	Adapted tool but not reported. Pilot study 2 trial observation days >90% reliability.	>90% trial observations, reliability on 50% of data.	yes	Direct live and video	30 min obs. Obs. zones. 1 min intervals	>1
SOPLAY/OSRAC-P	Berg [49]	Activity levels 1–3, gender, environmental factors, time of day, supervision, accessibility, organisation, equipment.	Adapted–limited details.	Not reported	yes	No information	2268 obs. Group time sampling.	Not reported
SOCARP	Foweather [58]	PA 1–5, supervisors, equipment, temperature. Group size, activity type, social interactions.	Adapted tool for play behaviours. Observer training conducted.	>80% inter rater agreement	yes	Video	5 min individual child obs. Time sampling technique 10 s obs. 10 s recording.	2
OSRAC-P	Bjørgen [38]	PA 1–5, activity type, activity context, environmental context, teacher/adult behaviour, time of day.	Adapted tool to use PA levels.	Independent coding compared and regulated.	yes	Video	50 h obs. Individual child observed every 15 s within 2 min.	2
OSRAC-P	Connelly [53]	PA 1–5, activity type, activity context, environmental context, teacher/adult behaviour, time of day.	Trained two observers until Kappa coefficient > 0.80 and maintained over three consecutive days.	Inter observer agreement Kappa coefficient > 0.80. 11.7% simultaneously and independently by two observers.	yes	Live direct	Individual child obs. >2 h. Momentary time sampling 5 s obs. 25 s recording.	2
OSRAC-P	True [72]	PA 1–5, activity type, activity context, environmental context, teacher/adult behaviour, time of day.	Not reported.	Not reported	yes	Live direct	Individual child obs. >5 h. 600 30 s intervals per child.	Not reported
OSRAC-P	Hustyi [59]	PA 1–5, activity type, activity context, environmental context, teacher/adult behaviour, time of day.	Not reported.	Reliability of observer agreement, 99%, 96%, 98%, 98%.	yes	Video	Individual child obs. Continuous 5 s partial-interval.	2
OSRAC-P	Sando [63]	PA 1–5, activity type, activity context, environmental context, teacher/adult behaviour, time of day.	Modified to used PA levels only. Trained three researchers.	Inter rater agreement 92%, Kappa coefficient 0.65.	yes	Video	Individual child 858 obs. 2 min intervals, 6 min break. 12 min.	2
OSRAC-P	Nicaise [61]	PA 1–5, activity type, activity context, environmental context, teacher/adult behaviour, time of day.	Modified using CARS.	15 IOA checks, average scores across categories 85.4, 87.3, 87.8, 81.4, 97.5%	yes	Live direct	Individual child 204 obs. 15–30 min, 5 s observation/25 s recording interval.	4
OSRAC-P	Nicaise [62]	PA 1–5, activity type, activity context, environmental context, teacher/adult behaviour, time of day.	Modified to measure PA levels, type, play context, group compositions, location.	15 IOA checks across categories 85.4, 87.3, 87.8, 97.5. Post: 80, 91.7, 93.9, 93.6.	yes	Live direct	Individual child 214 obs. 15–30 min, 5 s observation/25 s recording interval.	4
Categories of play	Torkar [70]	Two categories selected based on observations: Function play and play with rules. Social category recorded.	Based on Luchs and Fikus [71] forms of play and social categories.	Observer trained but no information reported.	no	Live direct	Individual child 50 obs. 25 min obs. period, every 4 min target child obs. Each play episode coded.	1
Categories of play	Sando [63]	Functional play, constructive play, symbolic play, mixed play, non-play, talking.	Based on Luchs and Fikus [71], Dyment and O’Connell [64], Fjortoft [37].	Coded by one observer and 10% reviewed by second researcher.	no	Video	Continuous coding. Individual child 858 obs. 2 min intervals, 6 min break. 12 min each child.	2
Categories of play	Veiga [73]	Fantasy play, role play, exercise play, rough and rumble, other play.	Based on Lindsey and Colwell [74]. 40 h of training.	80% level of inter-coding agreement. 27% videos double coded. Reliability Kappa coefficient = 0.81.	no	Video	Individual child 461 obs. 30 min obs. 3 min recordings. One-zero-time sampling 15 s intervals.	2
Categories of play	Bundy [51]	Categories of play: play, non-play. Social interactions, alone.	Not reported.	Trained rater checked by second rater via random selection of approx. 33%. Interrater reliability near perfect.	no	Video	15 min obs. at individual child level.	2
Categories of play	Brussoni [50]	Study-specific codes: prosocial behaviour, antisocial behaviour, channel surfing, child–teacher interactions, play with natural materials, risky play, gender segregated play.	Study-specific coding and based on Ladd, Price and Hart [83]; Pepler, Craig and Roberts [84]; Sandseter [85].	Cohen’s κ > 0.79.	no	Video	1971 min obs. coded 11,825 intervals. Individual child obs. 30 min play session, 10 sec intervals.	2
Categories of play	Dankiw [56]	Play behaviours, 23 codes, 5 domains: social interaction, social activity, cognitive activity, physical and motor skill activity, other.	Previous reliability testing by the research team. Based on Tranter and Malone [57].	Inter-rater reliability: 70% codes had ICC > 0.75. Inter-rater reliability 52% codes >	no	Live direct	964 min obs. Individual child. 3 × 20 min obs. 2 min intervals.	1
Taxonomy of affordances	Storli [67]	Categories of Heft’s [68] functional taxonomy of potential affordances.	Heft’s [68] functional taxonomy of potential affordances related to actualised affordances.	Not reported.	no	Both	Individual child obs.	2
Categories of actions	Sumiya [69]	Categories: locomotion, climbing, manipulation, sedentary, cycling, sloping, sand play, waterplay, sporting, horseplay.	Not reported.	Inter-rater agreement 0.91. Second coder coded 25% total time.	no	Video	97 min before, 87 min post alteration. Coded duration of actions and location at individual child level.	2
Categories of actions	Watts [75]	Time and frequency of engagement in play events.	Not reported.	Not reported.	no	Video	600 min obs. Individual child. 5 s min play event.	Not reported

Abbreviations: CARS, Children’s Activity Rating Scale; SOPLAY, System for Observing Play and Leisure Activity in Youth; SOCARP, System for Observing Children’s Activity and Relationships during Play; OSCRAC-P, Observational System for Recording PA in Children-Preschool; PA, Physical activity; Obs, observation(s).

## 4. Discussion

The aim of the current study was to examine the relationship between the ECE outdoor environment, PA, MC, and social interactions of children aged 3–7 years. The secondary aim was to identify previous observational tools and methods of measuring PA behaviours in the context of the physical and social environment. To the best of our knowledge, this is the first review to systematically synthesise evidence for all three outcomes in context of the ECE outdoor environment. Moreover, this review synthesises previous methods used to capture children’s behaviour in context of their social and physical environment. The key findings of this review support the notion that high-quality outdoor spaces comprising diverse portable affordances promote PA, social interactions, and cooperative play in young children. In this review, when children were physically active, they were more likely to be socially interacting in small groups. Based on the available, but limited evidence, MC was higher in children that had access to larger outdoor areas and when engaged in small group activities. Teacher interactions were negatively associated with PA in all but one study, where smaller groups and higher teacher to child ratios were associated with higher PA levels. These findings mostly align with previous systematic reviews, but also highlight several inconsistencies concerning associations with features of the outdoor play space and the impact of teacher interactions on children’s PA.

To address the secondary aim, this review reveals a high degree of variability in study designs, procedures and observational tools, emphasising the need for better consistency and standardised approaches in future related research. This review also reveals the limited evidence available from RCT and longitudinal studies, with most studies adopting uncontrolled cross-sectional designs.

### 4.1. Characteristics of the Outdoor Environment Affording Physical Activity

Based on the effect direction plot and associations, this review supports the notion that the quality of ECE outdoor play space is associated with PA levels of young children during outdoor free play. It also supports the case for targeting outdoor spaces as an effective intervention for decreasing sedentary time and increasing daily PA levels in young children. The findings from this review show that ECE outdoor provision can be enhanced by the presence of multiple portable affordances thus inviting actions that promote movement and physical active play. Based on the available evidence, interventions do not have to be expensive to be effective, as demonstrated in one study [51] which introduced recycled materials to the ECE play space. Further, simple reconfigurations to increase the availability of affordances in existing outdoor spaces were shown to increase PA, as demonstrated in several studies in this review [62,66,69]. Cross-sectional studies provide further evidence in support of loose portable affordances for mobilising young children, together with open spaces, tracks, and grassy landscapes. These findings are consistent with other conceptually similar systematic reviews; for example, Terrón- Pérez et al. [22] identified 10 studies that positively associated portable play equipment with PA. Despite these promising findings, intervention studies in this review did not account for the novelty factor in their study designs, and although interventions were conceptually similar, the outdoor contexts in which they were applied were not standardised. Therefore, it is difficult to draw absolute and firm conclusions given the high degree of contextual variability across studies.

In this review, three intervention studies used natural features to enhance the ECE play space, and three controlled cross-sectional studies used the natural environment as a comparator setting. When traditional ECE playgrounds were compared with natural settings, findings generally favoured the natural setting for promoting PA [38,67,70]. However, only one study yielded statistical significance which indicated a decrease in sedentary time [67]. One reason for this might be that studies had small sample sizes (< 50) and were perhaps underpowered to detect the differences between conditions. It should also be noted that the nature-based controlled cross-sectional studies in this review had the lowest MMAT scores (3*), indicating weak study designs. Intervention studies that introduced natural features found significant increases in TPA [55] and MVPA [51], but in contrast, Brussoni et al. [50] found a significant decrease in MVPA. This aligns with the broader literature which proposes that nature play may promote movements that are not necessarily physically intense [25]. For instance, natural features may afford different play activities, such as ‘risky play’ [50], which requires more careful considered movement resulting in lower PA intensities. Conversely, when associations are examined across all studies, certain nature-based features are consistently associated with higher PA levels, such as grassy areas, slopes, and open spaces [52,53,61,63,66,69], these findings are congruent with the previous literature and reinforce the importance of access to outdoor spaces for this age group. Despite inconsistent evidence in support of natural features for promoting PA, this review reinforces the notion that nature play may promote other under explored outcomes such as MC that may be a more theoretically matched outcome. Future research should consider capturing wider physical outcomes that may be more suited to the mechanisms of nature play. Several studies in this review explored the spatial layout of ECE outdoor play spaces by examining associations or reconfiguration effects on PA [52,66,69]. Findings indicate that PA behaviours are influenced by the spatial relationships between distinct zones of a play space. For instance, Sumiya et al. [69] reported that play and PA behaviours were altered by introducing a climbable mound, tires, and by removing equipment to create open space for games. Interestingly, PA was further increased in the sand box area due to carrying utensils becoming more accessible, hence children were more active transporting materials as opposed to sitting and moulding sand. Further, Smith et al. [66] reported that the centrality and adjacency of play settings may promote PA behaviours in children, perhaps due to the motivating effect of being able to see other children active in the surrounding areas. Notably, Clevenger et al. [52] examined the PA behaviours of children in relation to location across the school day through novel spatiotemporal analysis. This work highlights the change in children’s preferences for locations of high and low PA from morning to afternoon, which was previously not examined. Indeed, Clevenger et al. [52] observed low levels of PA when children congregated on pathways positioned by perimeter fences of the school yard. In this example, children were stationary to observe the world outside of the school grounds at specific times of the day, illustrating the spatiotemporal nature of behaviour patterns. Future research should examine changes in children’s preferences and dispreferences for play locations and PA patterns across the day. This extension to our understanding would enable more targeted interventions that may be more effective during specific times of the ECE day. This finding highlights the limitations of comparing studies where observation samples have been taken across different periods of the ECE day.

Several studies in this review explored the spatial layout of ECE outdoor play spaces by examining associations or reconfiguration effects on PA [52,66,69]. Findings indicate that PA behaviours are influenced by the spatial relationships between distinct zones of a play space. For instance, Sumiya et al. [69] reported that play and PA behaviours were altered by introducing a climbable mound, tires, and by removing equipment to create open space for games. Interestingly, PA was further increased in the sand box area due to carrying utensils becoming more accessible, hence children were more actively transporting materials as opposed to sitting and moulding sand. Further, Smith et al. [66] reported that the centrality and adjacency of play settings may promote PA behaviours in children, perhaps due to the motivating effect of being able to see other children active in the surrounding areas. Notably, Clevenger et al. [52] examined PA behaviours of children in relation to location across the school day through novel spatiotemporal analysis. This work highlights the change in children’s preferences for locations of high and low PA from morning to afternoon, which was previously not examined. Indeed, Clevenger et al. [52] observed low levels of PA when children congregated on pathways positioned by perimeter fences of the school yard. In this example, children were stationary to observe the world outside of the school grounds at specific times of the day illustrating the spatiotemporal nature of behaviour patterns. Future research should examine changes in children’s preferences and dispreferences for play locations and PA patterns across the day, thus enabling more targeted interventions. This finding also highlights the limitations of comparing studies where observation samples have been taken across different time points of the ECE day and accounts for inconsistencies in findings.

### 4.2. Social Interactions, PA, and the Outdoor Environment

An environment that offers diverse portable affordances invites collaborative play and social interactions as demonstrated by multiple studies in this review (see Table 5). The environment and its’ properties can be seen to act as a ‘teacher’ [50], inviting a variety of movement actions and challenges through cooperative play. When engaged in such games, activity levels were high and motor skills were more developed [58], suggesting that the social dimension is an important, but under-explored factor in developing MC. Of relevance, Foweather’s et al. [58] study noted that motor skills were lower in children that were observed to be ‘channel surfing’ at recess and were least engaged in games. Channel surfing is a term coined by Herrington and Brussoni [86] to describe the behaviour of children not engaged in an activity or game but often seen to be transitioning between activities. Interestingly, although this behaviour may exhibit more locomotor activity, and children appear to be physically active, the consequences of short play durations may be less-developed motor skills [58]. Further, Veiga’s et al. [73] research noted that mean duration of social interactions predicted social competence, and that social competence was positively associated with exercise play. Although these findings suggest that social competence and interactions may be associated with physically active play, causal inferences cannot be determined as many studies were cross-sectional designs. However, findings signal that the social context may moderate movement behaviours, and future research should examine social factors in relation to children’s engagement in physically active games in the outdoor setting. This is particularly under explored in relation to motor skill development and should be in the scope of additional and new investigations.

In this review, social interactions included those between the teacher and child. In all five studies that reported teacher–child interactions, the findings indicated a negative association with children’s PA [50,53,55,61,66]. It is plausible that teacher interactions in recess, or unstructured playtime are mainly custodial in manner, and therefore, in the context of child-led play, there were minimal interactions overall. Interestingly, teacher interactions were observed to decrease when ECE outdoor spaces were renovated or reconfigured, suggesting that the enhanced environment promoted more child-led play and less need for teacher intervention. This was also reflected in cross-sectional studies where affordance rich environments promoted longer play episodes and less teacher presence [50,55,60]. This finding supports the notion that given the right physical and social environment, children of this age group are intrinsically active, and are engaged by environments rich in portable affordances that facilitate diverse play [2,87]. Congruent with the broader literature, this review found that outdoor play spaces rich with natural features enabled more social interactions, cooperative play, and longer play episodes [27,87,88,89,90]. It is plausible that these environments are catalysts for encouraging open ended play and challenges involving risk and problem-solving skills which are exciting and thrilling for young children [91]. Given that play promoting environments are a likely mechanism for encouraging PA and social interactions, there needs to be a greater research emphasis on understanding the role of teachers as a moderating factor. Furthermore, as studies in this review represent eight countries, it is critical to consider the different cultural contexts and ECE policies regarding the supervision of outdoor play. Further investigation of cultural influences would help to illuminate how varying beliefs, practices, and policies shape the opportunities and barriers to outdoor play and PA in ECE settings. This would provide a more comprehensive understanding of how to design interventions and support teachers in fostering active play across diverse cultural contexts.

### 4.3. Motor Competence

Only three studies in the current review measured MC in relation to social and environmental factors. Aligning with previous studies examining the associations between playground size and PA [22,92], True et al. [72] found a positive association for total MC. Given the predictive association between PA levels and MC, and the previously reported association between PA and outdoor space, it is perhaps unsurprising to find benefits for developing the motor skills of this age group. This finding reinforces the importance of providing accessible outdoor spaces for preschool children’s physical development. Where gender differences were reported, the MC scores of boys were significantly higher than girls, and this finding reflects the wider body of literature [93]. It would be interesting to observe if these differences exist in preschools where the ECE outdoor play spaces are nature-based, as several studies in this review reported more mixed gender play under these conditions [38,60]. Affordances in the form of playground floor stencils was not an effective intervention to significantly improve MC scores in this age group [76]. This contrasts with the wider literature [94,95], which reports significant increases in PA resulting from similar interventions [95], which theoretically may have led to improvements in MC. However, previous studies did not measure MC; furthermore, children were older than the sample reported in this review [76]. By comparison, Foweather et al. [58] revealed that playground activity in the form of games without equipment was associated with higher total MC scores and locomotor scores, perhaps indicating that small group games that are child-initiated generate practice conditions conducive to developing motor skills. As previously mentioned, children that were alone in this context demonstrated lower MC scores, indicating that peer interaction is required to promote challenging practice conditions. Given that only three of the studies in this review measured MC in relation to social and environmental factors in the ECE outdoor play space, further research is warranted to understand children’s engagement with peers and the environment that positively drives the development of MC.

### 4.4. Measurement

This review identified a high degree of variability in observational tools and procedures used to analyse children’s behaviour in the ECE outdoor context. It is apparent that a consensus is required to establish standardised procedures for conducting systematic observational research in ECE outdoor settings for this age group. Further, there is a need to establish a valid and reliable observational tool to measure types of outdoor play in parallel with PA intensities in preschool aged children. This review highlights the potential value of integrating technologies to provide a more comprehensive overview of children’s PA and movement behaviours. For instance, Clevenger et al. [52] demonstrates the benefit of integrating accelerometer devices with GPS devices allowing spatiotemporal analysis of preschoolers’ PA across the school day. This work [52] illustrates the dynamic nature of children’s PA levels and location preferences throughout the ECE day. Thus, it confirms the problematic nature of comparing location-specific data across different time points. Finally, this review has revealed a gap in the literature as few studies assessed MC as an outcome measure despite reporting observations of motor skills during ECE outdoor play. In agreement with Foweather et al. [58]., further research examining the influence of ECE outdoor environmental features and active play on MC is warranted.

Among the studies included in this review, four validated observational tools (OSRAC-P, SOCARP, SOPLAY and CARS) were used to assess children’s PA, this suggests that there is a lack of consensus among researchers regarding the most suitable tool to evaluate ECE outdoor-based PA in young children. Furthermore, given that nine studies used additional categories of play, it would seem there is a need for the development of a validated tool to measure outdoor situated play in young children alongside PA measures [96]. Our recommendation for future research would be to adopt and further validate the tool for observing play outdoors [97], a recently developed tool based on several categories of play identified in this review [64,71,74]. This development would allow researchers to examine the relationships between PA levels and children’s play behaviours in an outdoor setting using standardised approaches, thus enabling the comparison of findings. Similarly, as there was considerable variability in the number of days assessed, total number of observations, and sampling techniques, this further emphasises the need for researcher agreement on observational protocols to examine children’s outdoor play behaviours. Based on our review, we recommend that future research should adhere to validated protocols, thus allowing researchers to compare outcomes across studies to establish a more robust evidence base for practitioners, educators, researchers, and policy makers.

In agreement with the literature, this study recommends that future research should use and report observation data in combination with technological devices such as GPS and accelerometers to reliably assess children’s behaviours in the context of their physical and social environment [24,25,98,99]. It is acknowledged that combining systematic observation with other methods enables a deeper understanding of interactions between the child, the environment, and social factors. This contextual information is needed to establish causal pathways [25,99] and is of relevance when attempting to identify what types of activities are afforded by features of the outdoor environment. As identified in the current study, MC as an outcome measure was underexplored and given the importance of MC for establishing positive health trajectories, this outcome should be examined in future studies alongside PA and contextual data.

This review identifies the benefits of integrating technologies to examine the effect of time alongside spatial PA behaviours. Thus, revealing the potential for research to employ these techniques to further understand how to optimally programme outdoor play periods across the ECE day. Furthermore, findings from spatiotemporal analysis highlight the potential source of inconsistencies in the literature, pertaining to areas of high and low PA. Further research examining both spatial and temporal components is therefore warranted, providing a clearer picture of young children’s preferences for play equipment or locations during different time periods of the day.

### 4.5. Limitations and Strengths

Firstly, as research in this field is limited, all study designs and qualities of evidence were included to provide a broad and comprehensive overview of the literature. Despite this, it is noteworthy that most of the included studies (*n* = 18/23) were of high quality (4/5 stars). Secondly, this review identified variability in observational tools and procedures, thus challenging the comparability between studies which emphasises the need for standardised approaches in future research designs and in reporting protocols. Thirdly, there was a high degree of variability between the depth of descriptions of the outdoor environment and the outdoor contexts themselves, again making comparability between studies difficult. Fourthly, although PRISMA recommend screening by two independent authors, only one author screened titles and abstracts in the current review. A final limitation of this review is the restriction of the date range to studies published between 2010 and the present, which may have excluded earlier research. However, this decision was made to focus on studies utilising more recent methodologies, technologies, and designs, ensuring the findings are reflective of contemporary practices and contexts.

The strengths of this review include following recognised and robust methodological procedures such as PRISMA, Cochrane’s recommendations for conducting synthesis without meta-analysis, and registering with PROPOSERO. Further, this review followed MMAT guidelines, an established procedure to appraise the quality of included studies. This systematic review provides comprehensive evidence synthesis on the characteristics of ECE outdoor environments in relation to children’s PA, MC, and social interactions. To the best of our knowledge, this is the first review to systematically synthesise evidence for all three outcomes in context of the ECE outdoor environment. Moreover, this review synthesises previous methods used to capture children’s behaviour in context of their social and physical environment. Key findings indicate the need for standardised integrated procedures to further the quality and depth of research in this area.

### 4.6. Practical Implications

Key findings from this review provide rich information for commissioners, stakeholders, practitioners, and researchers alike. The practical applications emerging from this research study are as follows:

- Enhance outdoor areas with portable affordances. Provide loose, adaptable, moveable play equipment, or recycled materials or tools.
- Incorporate natural elements, grassy areas, slopes and open spaces to encourage diverse forms of play.
- Optimise spatial layouts and play zones to allow for visibility and adjacency to foster physical and social activity.
- Provide low-cost interventions such as reconfiguring existing spaces or adding inexpensive portable affordances that can be effective mobilisers, without requiring large budgets.
- Encourage child-led play, minimise custodial teacher interactions during unstructured outdoor play.
- Teachers should focus on creating and maintaining affordance rich outdoor environments rather than directly intervening in periods of unstructured play.
- Facilitate small group activities and promote cooperative play to enhance engagement and intensity.

These findings will be shared through national and regional physical activity networks such as active partnerships. The current study has also identified further gaps in the research that relate to the development of MC through engagement with ECE outdoor spaces, and the role of social interactions in this context. This represents an opportunity for the motor development field and as such, findings from this review may be of interest to organisations such as the International Motor Development Research Consortium (IMDRC) [100]. Thus, providing opportunity for researchers, academics, and practitioners to convene and advance the knowledge base in this field.

## 5. Conclusions

This review supports the notion that ECE outdoor environments can effectively promote PA and social interactions in young children by offering outdoor spaces rich in diverse portable affordances and features that invite active play. Findings suggest that relatively simple and cost-effective interventions can promote PA and cooperative play behaviours such as introducing natural elements and/or reconfiguring affordances in an outdoor setting. Furthermore, these interventions reduced teacher involvement in children’s play, and children engaged in higher levels of PA when teacher interactions were low. In this review children were most active and were more likely to have developed MC when engaged in games and activities that were child initiated and in small groups. Despite this evidence, there are other underexplored potential moderating or mediating factors, such as time of outdoor play during the ECE day, or the specific nature of practitioner and peer interactions in outdoor settings. Findings also reveal the limited evidence examining children’s social interactions and MC, future studies should explore these behaviours in relation to motor skill development. Furthermore, it is difficult to ascertain whether motor skill development benefits from an affordance rich outdoor setting, but acknowledging the association between PA and MC, it is likely that MC will also benefit. Further research should examine the complex interplay of children’s behaviours in the ECE outdoor setting in relation to the development of MC alongside PA and play behaviours. This will require the development of standardised tools and integrated technologies to account for these complexities, and to further our understanding of optimum outdoor play spaces for child development. Furthering this evidence base will better inform policy, research, and practice in relation to play space design and interventions to increase high-quality movement time in ECE environments.

## Figures and Tables

**Table 1:** Search terms for PubMed.

[“green spaces”[tiab] OR “green space “[tiab] OR “beach”[tiab] OR “garden”[tiab] OR “outdoor environment”[tiab] OR “landscape”[tiab] OR “physical environment”[tiab] OR “natural environment”[tiab] OR “outdoor spaces”[tiab] OR “outdoor play “[tiab] OR “green space”[tiab] OR “green spaces”[tiab] OR “Space, Green”[tiab] OR “parks “[tiab] OR “outdoor learning”[tiab] OR “environment design”[tiab] OR “childcare centers”[tiab] OR “naturescape”[tiab] OR “playground”[tiab] OR “nature elements”[tiab] OR “natural elements”[tiab] OR “nature-based”[tiab] ] AND [“Child, Preschool”[mesh] OR “education”[mesh] OR “early years”[tiab] OR “EYFS”[tiab] OR “preschool”[tiab] OR “preschool children”[tiab] OR “preschool child”[tiab] OR “early childhood education”[tiab] OR “nursery”[tiab] OR “early learning”[tiab] OR “kindergarten”[tiab] OR “childcare”[tiab] OR “daycare”[tiab] OR “pre-school”[tiab] OR “child*”[tiab] OR “forest school”[tiab] OR “nature school”[tiab] OR “forest kindergarten”[tiab] OR “nature preschool”[tiab] OR “forest preschool”[tiab] OR “outdoor preschool”[tiab]] AND [“Motor Skills”[mesh] OR “Child Development”[mesh] OR “Exercise”[mesh] OR “social interactions”[mesh] OR “social behaviour”[mesh] OR “physical activity”[tiab] OR “motor development”[tiab] OR “motor competencies”[tiab] OR “fundamental movement skills”[tiab] OR “affordances”[tiab] OR “foundational movement skills”[tiab] OR “motor skills”[tiab] OR “movement”[tiab] OR “movement behaviour”[tiab] OR “movement behaviour”[tiab] OR “physical literacy”[tiab] OR “social interactions”[tiab] OR “social interaction “[tiab] OR “social context”[tiab] OR “social functioning”[tiab] OR “social outcomes”[tiab] OR “physical outcomes”[tiab]]

**Table 2:** Summary of study characteristics.

Author, Publication Year and Country	Sample Size	Mean Age or Range (Years)	Study Aim	Study Design	MMAT	Outdoor Intervention/Exposure and Environment Quality Measure	Outcome Measures
Berg (2015), Canada [49]	4 ECE centres	3–5 years	To determine the relationship between playground environments and quantity of young children’s PA levels.	Uncontrolled cross-sectional	5*	Observations of four different ECE outdoor play spaces. Features of each centre described.	Systematic Observation using SOPLAY and OSRAC-P to measure PA.
Bjørgen (2016), Norway [38]	24	3–4 years	The aim was to examine how affordances in two outdoor environments explain children’s level of PA.	Controlled cross-sectional	3*	Comparison of Kindergarten’s outdoor playground vs. natural environment. Herrington’s 7 Cs assessed.	Systematic Observation using OSRAC-P to measure PA and social interactions.
Brussoni (2017), Canada [50]	45	4.28 ± 0.63	To examine the effects of a Seven Cs’ design intervention to increase access to nature and risky outdoor play opportunities on children’s play, social, mental health, and PA.	Uncontrolled before and after	5*	Renovation: Herrington et al. Seven Cs’ criteria for outdoor play design. Increase access to nature and risky play opportunities.	Systematic Observations of play and social interactions. ActiGraph accelerometers to measure PA.
Bundy (2017), Australia [51]	221	6 ± 0.6	To assess a simple intervention to increase children’s PA, play, perceived competence/social acceptance, and social skills.	Cluster RCT	5*	Introduction of recycled loose parts to the ECE playground.	Systematic Observations of play and social interactions. ActiGraph accelerometers to measure PA.
Clevenger (2020), USA [52]	34	2–5 years	Purpose to use a spatiotemporal approach to hot spot analysis to identify where and when clusters of PA occur on the playground.	Uncontrolled cross-sectional	5*	ECE playground descriptions, GPS and ArcMap to conduct spatiotemporal analysis.	ActiGraph accelerometers to measure PA. Qstarz GPS, ArcMap and observations to measure locations or activity.
Connelly (2021), Canada [53]	30	4.5 ± 0.05	To measure PA levels of children and identify factors related to MVPA in the outdoor context.	Uncontrolled cross-sectional	5*	Observations of ECE centres mapped zones in relation to activity types and levels.	Systematic Observation using OSRAC-P to measure PA.
Cosco (2010), USA [54]	53		Analysis of two ECE centres playgrounds mapping location to PA.	Uncontrolled cross-sectional	5*	Comparison of kindergarten’s outdoor spaces using behaviour mapping GIS.	Systematic Observation using CARS to measure PA and GIS behavioural mapping.
Cosco (2014), USA [55]	804	4–5 years	To evaluate the effectiveness of preventing obesity by design, a childcare centre renovation intervention.	Uncontrolled before and after	4*	Renovation: Improved environmental quality. Preschool Outdoor Environments Measurement Scale	Systematic Observation and behavioural mapping using CARS to measure PA, location, social interactions.
Dankiw (2023), Australia [56]	17	3–5 years	To describe where and how children play in outdoor early childhood settings.	Uncontrolled cross-sectional	5*	Characterisation of play spaces. Nature vs. manufactured play zones.	Systematic Observation and behavioural mapping (Tranter & Malone, 2004 [57]) to measure play, social, physical, and motor skill activity.
Foweather (2021), UK [58]	133	4.7 ± 0.5	To examine the associations between play behaviours and FMS during recess at preschool.	Uncontrolled cross-sectional	5*	Observations of 12 preschools outdoor play behaviours in relation to activity type and equipment.	Systematic Observation using SOCARP to measure play behaviours. CMPS motor skill protocol to measure foundational movement skills.
Hustyi (2012), USA [59]	4	4	To assess the effects of environmental context on the level of PA in children.	Descriptive	3*	Observation and comparison of three outdoor contexts, with or without artifacts.	Systematic Observation using OSRAC-P to measure PA.
Moreira (2022), Portugal [60]	26	4.8 ± 0.78	Examine the relationship between kindergarten’s outdoor environment and preschoolers’ social functioning.	Controlled cross-sectional	4*	Comparison of low vs. high quality. High-quality characteristics: sandbox, loose parts, bench, trees, swing and portable slide. Children’s Physical Environments Rating Scale	Peer social proximity Radio Frequency Id Devices to measure social interactions.
Nicaise (2011), USA [61]	51	4.25 ± 0.52	Analysis of two ECE centre playgrounds mapping location to PA.	Uncontrolled cross-sectional	5*	Observations of ECE centre mapped zones in relation to activity types and levels.	Systematic Observation using OSRAC-P to measure PA.
Nicaise (2012), USA [62]	50 baseline, 57 postintervention	4.7 baseline, 4.3 postintervention	To examine the effect a physical reconfiguration and repurposing of a playground’s spaces have on children’s PA.	Uncontrolled before and after	5*	Reconfiguration playground space based on Louv’s urban naturalism concepts.	Systematic Observations of mapped zones using OSRAC-P and ActiGraph accelerometers to measure PA, location and social interactions.
Sando (2020), Norway [63]	73	4.2	Aim to develop knowledge about play episodes where children experience high well-being and PA in the outdoor environment and how children utilise affordances in these situations.	Uncontrolled cross-sectional	5*	Observation of ECE outdoor play spaces, categories for observing places devised from previous research (Cosco et al., 2010 [54]; Dyment & O’Connell, 2013 [64]; Lerstrup & van der Bosch, 2017 [65]).	Systematic Observation using OSRAC-P to measure PA, social interactions and play.
Smith (2016), USA [66]	30 ECE centres	3–5 years	To examine the associations between adjacency, centrality and clustering on children’s PA levels.	Uncontrolled cross-sectional	5*	Observations of ECE outdoor play spaces using behavioural mapping GIS.	Systematic Observation using CARS to measure PA with GIS for behavioural mapping.
Storli (2010), Norway [67]	16	3–5 years	Explore children’s physically active play outdoors in a traditional playground and natural (nature) environment.	Controlled cross-sectional	3*	Traditional playground vs. natural environment. Heft’s (1988) [68] functional taxonomy.	Systematic observation of features and play. ActiGraph Accelerometers to measure PA.
Sumiya (2021), Japan [69]	6	5.08 ± 0.2	Does the alteration of spatial layout of playground affects the pattern of play activity and PA levels of young children in the playground?	Uncontrolled before and after	5*	Reconfiguration to make available affordances for climbing easy access to toys and objects.	Systematic Observations of mapped zones and actions observed, ActiGraph accelerometers to measure PA.
Torkar (2017), Slovenia [70]	25	4–5 years	The aim was to investigate children’s play activities and PA on a traditional playground and on a forest (natural) playground.	Controlled cross-sectional	3*	Traditional vs. forest (natural) playground.	Systematic observations of play (Luchs and Ficus, 2013 [71]). GPS.
True (2017), USA [72]	229	4.2 ± 0.6	To examine the contribution of various preschool environmental characteristics to children’s locomotor, object control, and total gross motor scores.	Uncontrolled cross-sectional	5*	ECE outdoor playgrounds rated Early Childhood Development Rating Scale Revised (ECERS-R).	Systematic Observation using OSRAC-P to measure PA. CMPS to measure motor competence.
Veiga (2017), Portugal [73]	73	4.6 ± 0.3	Examine social play, competence, and playground interactions	Uncontrolled cross-sectional	5*	Observations of ECE playground, types of play and social interactions and competence	Radio Frequency Id Devices to measure social interactions. Systematic Observations of play behaviours based on Lindsey and Colwell (2013) [74].
Watts (2022), USA [75]	36	3–4 years	Explore the differences in children’s playtime engagement in facilitating gross motor skill development between nature-based versus traditional manufactured equipment.	Uncontrolled before and after	2*	Renovation: Manufactured vs. natural ECE playground. Mapped zones of outdoor play space.	Systematic Observations of play events
Webster (2023), USA [76]	51	4.3 ± 0.6	The aim was to test the effectiveness of a playground stenciling intervention to increase FMS, PA and reduce time spent sedentary among preschoolers attending ECE centres.	RCT	4*	ECE playground stencils. Nutrition and PA in Childcare Survey	Systematic Observation using SOPLAY and ActiGraph accelerometers to measure PA.

Abbreviations: ECE, early childhood education; PA, physical activity; FMS, fundamental movement skills; USA, United States of America; UK, United Kingdom; RCT, randomised controlled trial; MMAT, mixed-methods appraisal tool; SOPLAY, system for observing play and leisure activity in youth; CARS, children activity rating scale; OSRAC-P, the observational system for recording activity in children-preschool; SOCARP, system for observing children’s activity and relationships during play; CMPS, children’s activity and movement in preschool study motor skills protocol; GPS, global positioning system; GIS, geographic information system.

**Table 4:** Relationship between features of the ECE outdoor environment and PA.

Outdoor Features	Study ID	Negative Association	Null	Positive Association
Mulch, hard surface	Clevenger, Smith	1 [52]	1 [66]	
No. adjacencies, centrality	Smith			1 [66]
Outdoor space size	Smith			1 [66]
Density	Smith			1 [66]
Open space/grassy areas	Smith, Connelly, Clevenger, Nicaise, Nicaise, Sando, Sumiya		2 [63,66]	4 [52,53,61,62,64,69]
Grassy hill	Nicaise			1 [62]
Natural elements	Sando, Brussoni, Nicaise	2 [50,63]		1 [62]
Balls, frisbees, tires	Connelly, Nicaise, Smith, Sumiya			4 [53,61,66,69]
Loose parts	Bundy, Smith			2 [51,66]
Toys	Sando	1 [63]		
Wheeled toys	Smith, Connelly, Cosco, Nicaise, Sando		2 [61,63]	3 [53,55,61,66]
Fixed equipment	Clevenger, Connelly, Nicaise, Sando	2 [52,62]		4 [52,53,61,63]
Playground	Nicaise, Nicaise			2 [61,62]
Playground markings	Webster		1 [76]	
Wood step/slope	Sumiya			1 [69]
Sandbox	Connelly, Clevenger, Sando, Sumiya, Smith	1 [52]	2 [63,66]	2 [53,69]
Cycle tracks/looped pathways	Cosco, Nicaise, Nicaise, Sando, Sumiya, Smith		1 [66]	5 [55,61,62,63]
Sand/mud	Sando		1 [63]	
Sidewalk	Clevenger	1 [52]		

Note: Associations reported *p* < 0.05.

**Table 5:** ECE outdoor environment, social interactions and physical activity.

Study ID	Study Design	Sample Size	Intervention/Exposure Concept	MMAT	Outcome Summary of Social Interactions and PA
Cosco [55]	UBA	804	Nature and affordance renovation	4*	Less peer interaction associated with increased activity (*p* < 0.05). Teacher interactions negative effect on PA (*p* < 0.05). Less teacher presence positive effect on PA (*p* < 0.05). Less teacher presence post renovation (*p* < 0.05). Girls more sedentary and 38.9% less engaged in MVPA.
Brussoni [50]	UBA	45	Nature and risk affordances	5*	Reduced teacher interactions at centre with more affordances (*p* < 0.001). More diverse play at centre with more affordances after intervention (*p* < 0.001). Gender segregated play did not change.
Webster [76]	RCT	51	Affordances	4*	Mostly child led in small groups 3–4. Children created games to incorporate stencils.
Bundy [51]	Cluster RCT	221	Affordances	5*	Increase in play and social interactions (d = 0.27).
Moreira [60]	CCS	26	High vs. low environmental quality	4*	High quality kindergarten: children more time with peers (2 children), less time alone and mixed gender (*p* < 0.05). More time with opposite gender (*p* < 0.05).
Torkar [70]	CCS	25	Traditional vs. nature	3*	More chasing games in forest compared to traditional playground. Boys more active than girls in the natural environment.
Dankiw [56]	CCS	17	Manufactured vs. natural	5*	More cooperative play and social interactions in nature play zones (*p* = 0.008). More exploratory play in the nature play zones (*p* = 0.002).
Bjørgen [38]	CCS	24	Traditional vs. natural	3	Increase cooperative play in natural environment. More physically active free play together in natural environment.
Storli [67]	CCS	16	Traditional vs. nature	3*	Consistent behaviours across both environments.
Clevenger [52]	UCS	34	ECE playground	5*	Location of clusters of high or low activity counts changed both within [intraperiod] and between outdoor periods (interperiod).
Smith [66]	UCS	6083	ECE playground	5*	Number of adjacencies and centrality associated with social interactions (*p* < 0.01). Teacher presence negatively associated with PA (*p* < 0.001). Peer child–child interactions positively associated with PA (*p* < 0.05). Boys with wheeled toys (*p* = 0.001) or interacting with peers (*p* = 0.05) exhibit more PA.
Nicaise [61]	UCS	51	ECE playground	5*	Children were more 2.1 times more likely to engage in MVPA when alone compared to child-adult play. Compared to peer groups, children were 1.6 and 1.3 times more likely to engage in MVPA when alone and with a single peer. Boys spent more intervals than girls in MVPA (*p* < 0.05).
Connelly [53]	UCS	30	ECE playground	5*	Children initiated activities in 89.5% intervals. Children were 1.59 times more likely to engage with MVPA when in a peer-only group compared to a context with adults present. Teacher-led activity was not associated with MVPA. Boys spent more intervals in MVPA than girls (*p* < 0.001).
Sando [63]	UCS	73	ECE playground	5*	High well-being and PA positively associated with being with other children (*p* = 0.003). Mixed play positively associated with PA and high well-being (*p* = 0.000). Positive association between high well-being, being a boy and PA (*p* = 0.043).
Berg [49]	UCS	4 centres	ECE playground	5*	Smaller class size to teacher ratio favoured more PA.
Foweather [58]	UCS	133	ECE playground	5*	Active games without equipment positively associated with FMS total scores (*p* = 0.01) and locomotor skills (*p* = 0.013). Time spent in locomotion (channel surfing) negatively associated with locomotor skills (*p* = 0.009).
Veiga [73]	UCS	73	ECE playground	5*	Mean duration of interactions positively associated with social competence (*p* = 0.032). Social competence positively associated with exercise play (*p* = 0.002). Boys engaged in more rough and tumble play than girls (*p* = 0.036). Girls engaged in more exercise play than boys (*p* = 0.048). Boys had more interactions and higher % time in interactions and larger group size than girls (*p* = 0.008, *p* = 0.040, *p* = 0.004).

Abbreviations: ECE, early childhood education; PA, physical activity; MMAT, mixed-methods appraisal tool; UBA, uncontrolled before and after; RCT, randomised controlled trial; CCS, controlled cross-sectional; UCS, uncontrolled cross-sectional.

**Table 6:** Relationships between ECE outdoor environment, social behaviours, and motor competence.

Study ID	Study Design	Outdoor Intervention/Environment	Method/Measures	MMAT	Outcome Summary
Webster [76]	RCT	ECE playground stencils. Nutrition and PA in Child Care Survey	Observation (SOPLAY), motor competence TGMD-3	4*	No significant differences in TGMD-3 between control and intervention group postintervention. Increase across all TGMD-3 in both intervention and control groups.
Foweather [58]	Uncontrolled cross-sectional	ECE playground.	Observations of play behaviours (SOCARP), Motor Skill Protocol (CMPS).	5*	Time spent in active games without equipment positively associated with total and locomotor FMS scores. Locomotor observations negatively associated with locomotor skills.
True [72]	Uncontrolled cross-sectional	ECE playgrounds.	Observation (OSRAC-P), motor competence (CMPS)	5*	Larger playgrounds associated with better motor competence scores. Boys had significantly higher total scores and object control than girls.

Abbreviations: RCT, randomised controlled trial; ECE, early childhood education; TGMD-3, test of gross motor development- 3rd edition; SOPLAY, System for Observing Play and Leisure Activity in Youth; SOCARP, System for Observing Children’s Activity and Relationships during Play; FMS, fundamental movement skills; CMPS, children’s activity and movement in preschool study motor skills protocol; OSCRAC-P, Observational System for Recording PA in Children-Preschool.

## Data Availability

No new data were created or analysed in this study. Data sharing is not applicable to this article.

## Appendix A

**Table A1:** MMAT quality assessment outcomes.

First Author	Qualitative Studies					Randomised Controlled Trials					Non-Randomised Studies					Quantitative Descriptive Studies					Mixed-Methods Studies					MMAT Quality	Comments
	1.1	1.2	1.3	1.4	1.5	2.1	2.2	2.3	2.4	2.5	3.1	3.2	3.3	3.4	3.5	4.1	4.2	4.3	4.4	4.5	5.1	5.2	5.3	5.4	5.5		
Berg [49]																1	1	1	1	1						*****	
Bjørgen [38]											1	1	0	0	1											***	
Brussoni [50]											1	1	1	1	1											*****	
Bundy [51]						1	1	1	1	1																*****	
Clevenger [52]																1	1	1	1	1						*****	
Connelly [53]																1	1	1	1	1						*****	
Cosco [54]																1	1	1	1	1						*****	
Cosco [55]											1	1	0	1	1											****	
Dankiw [56]																1	1	1	1	1						*****	
Foweather [58]																1	1	1	1	1						*****	
Hustyi [59]																0	0	1	1	1						***	
Moreira [60]											1	1	1	0	1											****	
Nicaise [61]																1	1	1	1	1						*****	
Nicaise [62]											1	1	1	1	1											*****	
Sando [63]																1	1	1	1	1						*****	
Smith [66]																1	1	1	1	1						*****	
Storli [67]											1	1	0	0	1											***	
Sumiya [69]											1	1	1	1	1											*****	
Torkar [70]											1	0	1	0	1											***	
True [72]																1	1	1	1	1						*****	
Veiga [73]																1	1	1	1	1						*****	
Watts [75]											1	0	0	0	1											**	
Webster [76]						1	1	1	0	1																****	

Study quality was assessed using the mixed-methods assessment tool (MMAT) and is reported using asterisks (*) to describe the quality rating of the article, ranging from 1*, where 20% of the quality criteria have been met, to 5*****, where 100% of the quality criteria have been met.
